# Induction of Cytoprotective Pathways Is Central to the Extension of Lifespan Conferred by Multiple Longevity Pathways

**DOI:** 10.1371/journal.pgen.1002792

**Published:** 2012-07-19

**Authors:** David E. Shore, Christopher E. Carr, Gary Ruvkun

**Affiliations:** 1 Department of Molecular Biology, Massachusetts General Hospital, Boston, Massachusetts, United States of America; 2 Department of Genetics, Harvard Medical School, Boston, Massachusetts, United States of America; 3 Department of Earth, Atmospheric, and Planetary Sciences, Massachusetts Institute of Technology, Cambridge, Massachusetts, United States of America; Stanford University Medical Center, United States of America

## Abstract

Many genetic and physiological treatments that extend lifespan also confer resistance to a variety of stressors, suggesting that cytoprotective mechanisms underpin the regulation of longevity. It has not been established, however, whether the induction of cytoprotective pathways is essential for lifespan extension or merely correlated. Using a panel of GFP-fused stress response genes, we identified the suites of cytoprotective pathways upregulated by 160 gene inactivations known to increase *Caenorhabditis elegans* longevity, including the mitochondrial UPR (*hsp-6*, *hsp-60*), the ER UPR (*hsp-4*), ROS response (*sod-3*, *gst-4*), and xenobiotic detoxification (*gst-4*). We then screened for other gene inactivations that disrupt the induction of these responses by xenobiotic or genetic triggers, identifying 29 gene inactivations required for cytoprotective gene expression. If cytoprotective responses contribute directly to lifespan extension, inactivation of these genes would be expected to compromise the extension of lifespan conferred by decreased insulin/IGF-1 signaling, caloric restriction, or the inhibition of mitochondrial function. We find that inactivation of 25 of 29 cytoprotection-regulatory genes shortens the extension of longevity normally induced by decreased insulin/IGF-1 signaling, disruption of mitochondrial function, or caloric restriction, without disrupting normal longevity nearly as dramatically. These data demonstrate that induction of cytoprotective pathways is central to longevity extension and identify a large set of new genetic components of the pathways that detect cellular damage and couple that detection to downstream cytoprotective effectors.

## Author Summary

Many mutations that increase animal lifespan also confer stress tolerance, suggesting that cytoprotective mechanisms underpin the regulation of longevity. It has not been established, however, whether the induction of individual cytoprotective pathways is essential for lifespan extension, or merely correlated. To establish whether the regulatory pathways for the induction of cytoprotective responses are key in the extension of lifespan, we performed an RNAi screen for gene inactivations that decouple the activation of cytoprotective pathways from xenobiotic stimuli that normally induce them. The screen identified 29 genes that constitute the regulatory cascades of the unfolded protein response, oxidative stress response, and detoxification. These upstream regulatory genes are critical to stress tolerance and the extension of lifespan conferred by decreased insulin/IGF-1 signaling, disruption of mitochondrial function, or caloric restriction, but have little effect on normal longevity.

## Introduction

Lifespan can be extended in *C. elegans* and other organisms by a variety of ostensibly deleterious interventions: disruption of mitochondrial function, disruption of translation, disruption of insulin/IGF-1 signaling, caloric restriction, exposure to xenobiotics and others. The counterintuitive benefits of these stressful stimuli suggest a hormetic mechanism rooted in the beneficial induction of cytoprotective pathways that respond to environmental challenges, such as starvation, heat, or exposure to xenobiotics. These cytoprotective pathways may represent the mechanisms that drive lifespan extension. While the correlation of stress tolerance and longevity is well established, the underlying cytoprotective pathways have not been fully explored.

Many of the gene inactivations that extend lifespan encode core, conserved components of cells, such as translation factors or mitochondrial proteins, many of which are the molecular targets of known xenobiotics [1]. Lifespan-extending inactivation of cytochrome C reductase, ATP synthase, F59C6.5 in electron transport chain (ETC) complex I, or cytochrome C oxidase may induce the same cytoprotective responses as the xenobiotics that target them, which include antimycin, oligomycin, rotenone and sodium azide, respectively. Similarly, a wide variety of xenobiotics disrupt translation, including hygromycin, genetecin and emetine. Disruption of endoplasmic reticulum (ER) function also extends longevity and may induce cytoprotective mechanisms effective against ER-targeted xenobiotics such as tunicamycin or thapsigargin. The parity of essential cell components targeted by xenobiotics and those that extend longevity upon inactivation suggests that long-lived animals engage cytoprotective mechanisms that evolved as cellular homeostatic and detoxification responses to xenobiotics and virulence factors produced by other organisms.

Cytoprotective responses, including chaperones, antioxidants and pathogen response genes, as well as xenobiotic detoxification mechanisms, can protect extant components of the cell and may contribute to lifespan extension. Genetic studies have identified over 50 mutations that extend the lifespan of *C. elegans*, and each is resistant to one or more stressors, such as oxidative damage, heat stress or irradiation [2], [3]. The oxidative stress theory of aging has driven extensive analysis of oxidative damage in particular, and while long-lived animals are resistant to compounds that generate ROS, such as paraquat, identification of underlying mechanisms has proven challenging [4], [5], [6], [7], [8], [9], [10], [11], [12]. Longevity is also correlated with thermotolerance, and expression of the heat shock response gene *hsp-16.2* predicts longevity in *C.elegans* [13], [14], [15], [16], [17], [18]. In a genetic screen for enhanced thermotolerance, the majority of isolated mutants were long-lived by at least 15% [19]. Other protein folding mechanisms, such as the ER and mitochondrial unfolded protein responses, contribute to longevity as well [20], [21], [22], [23]. Cellular damage may result from the production of toxic metabolic byproducts or exposure to xenobiotics, consistent with the extension of lifespan by overexpression of the detoxification transcription factor *skn-1*, a gene that is also required for lifespan extension in *daf-2* mutants [24], [25], [26], [27], [28], [29], [30], [31]. The potential influence of diverse cytoprotective functions on longevity is underscored by the heat, ROS and toxin resistance of long-lived animals.

Consistent with the stress-tolerant phenotypes of long-lived animals, cytoprotective mechanisms are activated in long-lived mutants. Disruption of insulin/IGF-1 signaling induces heat shock (*hsp-16.49*, *hsp-16.11*, *hsp-16.1*, *hsp-16.2* and *hsp-12.6*), antioxidant (*ctl-1*, *ctl-2* and *sod-3*), and pathogen response (*lys-7*, *lys-8* and *spp-1*) genes [32]. The long-lived mitochondrial mutants *isp-1*, *clk-1* and *cyc-1* induce the mitochondrial unfolded protein response [33]. Inactivation of the translation initiation factor *ifg-1* induces the transcription of 51 stress responsive genes, including *daf-16* and *skn-1* [34]. In each of these long-lived mutants, evidence suggests concurrent induction of detoxification mechanisms. The detoxification of xenobiotics in many systems, including *C. elegans*, involves the upregulation of cytochrome P450's (CYPs), UDP-glucuronosyltransferases (UGTs), and glutathione S-transferases (GSTs). Transcriptional profiling of the long-lived *daf-2* insulin/IGF-1 signaling mutant reveals *daf-16*-dependent upregulation of these functions [32], [35]. A xenobiotic response is similarly induced in long-lived mitochondrial mutants and lifespan extension by disruption of translation requires *skn-1*, which participates in xenobiotic stress tolerance [36], [37]. While the response to xenobiotics is, in part, the upregulation of detoxification, other cytoprotective mechanisms, such as chaperones, mitigate cellular damage; detoxification and cytoprotection may both be components of a xenobiotic response apparatus mobilized by various aging interventions.

Mechanistic evidence supports the causality of cytoprotective gene activation in lifespan extension. Loss of chaperone expression through inactivation of *hsf-1*, the transcriptional regulator of the heat shock response genes, abrogates lifespan extension in a *daf-2* mutant, while overexpression extends the lifespan of wild-type animals [38]. The ER unfolded protein response (UPR) underlies lifespan extension in *daf-2* mutants and in response to caloric restriction [22], [39]. The mitochondrial UPR is required for lifespan extension in the mitochondrial mutants *isp-1* and *clk-1* [21]. Lifespan regulatory factors, including *daf-2*, *hif-1*, *skn-1* and *hsf-1* are required for pathogen defense, further suggesting that these pathways coordinate critical elements of cytoprotection [40], [41], [42], [43], [44], [45], [46], [47], [48], [49]. These findings highlight the potential contributions of a range of cytoprotective pathways to lifespan extension, but a systematic genetic analysis of the regulation of cytoprotective mechanisms in diverse models of lifespan extension has not been conducted.

We tested the hypothesis that the regulation of cytoprotective gene expression in long-lived animals underlies lifespan extension across mechanistically diverse models of this phenotype. We utilized xenobiotic and genetic stimuli in an RNAi screen to identify regulatory genes required for the appropriate induction of *hsp-6*, *hsp-4*, *gst-4* and *sod-3* in long-lived animals. We directly addressed the activity of these cytoprotection regulatory genes in lifespan extension by inactivation and subsequent lifespan analysis in three functionally diverse long-lived mutants, *isp-1*, *eat-2* and *daf-2* (overview in Figure S1). We find that these cytoprotective regulatory genes are critical to lifespan extension in all three longevity backgrounds. These results provide mechanistic support for the hypothesis that lifespan extension occurs through the activation of cytoprotective pathways triggered by xenobiotic or genetic means.

## Results

### Identification of Regulators of Cytoprotective Gene Induction

Long-lived mutants express cytoprotective genes at elevated levels. To identify the cytoprotective pathways induced by the diverse conditions that confer lifespan extension, we analyzed the induction of 13 stress-responsive GFP fusion genes functioning in the response to heat, ER stress, mitochondrial stress, oxidative damage, pathogenesis, osmotic stress, xenobiotics, and decreased insulin/IGF-1 signaling (Table S1) by each of 160 gene inactivations found to increase longevity in high-throughput RNAi screens (Table S2) [1], [20], [23], [32], [50], [51], [52], [53], [54], [55], [56], [57]. Clustering the expression of p*hsp-6*::gfp (Mt UPR), p*hsp-60*::gfp (Mt UPR), p*hsp-4*::gfp (ER UPR), p*gst-4*::gfp (detoxification), p*sod-3*::gfp (ROS), pF55G11.7::gfp (pathogenesis) and p*gpdh-1*::gfp (osmotic stress) generates distinct groups of longevity gene inactivations (Figure S2). Overall, cytoprotective gene activation is a hallmark of the most potent lifespan extension mechanisms. The average mean lifespan extension amongst the 88 gene inactivations that induce at least one fusion gene (Figure S2) is 27.3%, while the 72 gene inactivations that do not activate a single fusion gene exhibit an average extension of 12.5% (t-test p = 7.7E−12) (Figure S2, Table S2) [1], [50], [51].

To discern how interventions that extend longevity couple to the activation of cytoprotective pathways, we sought to identify the genes required for the activation of *hsp-6* (Mt UPR), *hsp-4* (ER UPR), *sod-3* (ROS response) and *gst-4* (detoxification) by drugs or genetic triggers. Activation of cytoprotective genes requires the capacity to detect the disruption of an essential cell function, such as translation or mitochondrial function, and to generate signals that activate downstream responses. The mechanisms by which these events occur remain largely unknown. We reasoned that components of longevity signaling might emerge from an RNAi screen to identify gene inactivations that disrupt the induction of cytoprotective pathways. Because many gene inactivations that confer longevity extension encode targets of naturally occurring xenobiotics, analysis of toxin response may explore the same cytoprotective pathways activated in long-lived mutant animals. We raised *C. elegans* to young adulthood before treating the animals with toxins to induce the expression of longevity-correlated cytoprotective genes. Tunicamycin, an antibiotic that disrupts N-linked glycosylation in the ER, was employed for the activation of the ER UPR reporter p*hsp-4*::gfp [20]. Antimycin disrupts complex III of the electron transport chain, activating the mitochondrial UPR reporter p*hsp-6*::gfp. Sodium azide treatment activates the *skn-1* target p*gst-4*::gfp, and the activity of the *daf-16* target p*sod-3*::gfp was modulated via a temperature sensitive allele of *daf-2* [53], [58]. We used RNAi to screen for gene inactivations that blocked the expected cytoprotective response to each drug or genetic stimulus.

The screen encompassed ∼1500 gene-inactivating RNAi constructs, assayed for inhibition of each of the four cytoprotective responses. This library was composed of 395 kinases and 610 transcription factors, as well as two cherry-picked small RNA and longevity sublibraries. Because small RNA pathways have been implicated in stress responses, we screened 317 gene inactivations that have emerged from screens for defects in microRNA or RNAi functions [59], [60]. Gene inactivations that abrogate the increase in longevity conferred by low insulin/IGF-1 signaling have also been identified and potentially regulate cytoprotective functions. We therefore included this set of 179 gene inactivations as well [61].

The primary screen identified 73 gene inactivations required for appropriate activation of cytoprotective responses (Table 1, Table S3). Known stress response regulatory factors (*ire-1*, *skn-1*, *daf-16*) score strongly and each is highly specific to its known function (Table 1). Quantification of fluorescence intensity for 32 gene inactivations that scored most strongly in the primary optical screen confirmed the role of 29 genes in cytoprotective gene induction (Table 1). Induction of p*hsp-16.2*::gfp following heat shock and the expression of a non-stress-induced fusion gene, p*sur-5*::gfp, were quantified to control for generic transgene silencing phenotypes; none of these gene inactivations were potent transgene silencers (Table S4). Expression of the chromosomal loci corresponding to the screened fusion genes was analyzed by quantitative PCR to distinguish transgene dysregulation from regulation of the endogenous loci (Table S5). Results confirmed that the majority of these gene inactivations decouple the chromosomal cytoprotective loci from activation by toxins. While results were largely consistent, measured decreases in fluorescence from the GFP fusion genes were more dramatic than those detected by quantitative PCR for the corresponding genetic loci. The use of multi-copy transgenic constructs may contribute to this observation. Tissue specificity may do so as well, since quantitative PCR averages gene induction over all *C. elegans* cells while cytoprotective gene induction may be isolated to particular tissues. In addition, the efficacy of RNAi is reduced in neurons and other excitable cells.

**Table 1:** Gene inactivations that inhibit cytoprotective responses.

	p*hsp-4*::gfp	p*hsp-6*::gfp	p*sod-3*::gfp	p*gst-4*::gfp	
	tunicamycin	antimycin	*low ins. sig.*	azide	
Gene	Fluorescence (fold decrease)				Function
C06A8.2	**1.5**	**4.5**	ns	ns	Transcription; SNAPc
*rab-10*	**1.7**	**2.6**	ns	**1.8**	RAS GTPase; endocytosis
*pas-3*	**1.8**	**2.7**	**2.2**	ns	20S proteasome, regulatory subunit
*wnk-1*	**2.0**	ns	ns	ns	Serine/threonine protein kinase
*cpsf-4*	**2.3**	**3.9**	ns	ns	mRNA cleavage and polyA specificity
Y50D7A.11	**2.5**	**1.5**	ns	ns	Uncharacterized
F18F11.5	**2.6**	ns	ns	**2.4**	Serine/threonine protein kinase
*mdt-26*	**3.2**	**2.8**	ns	**1.5**	Transcription; mediator
F53F4.11	**3.6**	ns	ns	ns	Uncharacterized
*sptl-1*	**3.6**	**3.9**	ns	**2.8**	Sphingolipid synthesis
*cpsf-2*	**3.6**	**27.9**	**3.0**	**2.3**	mRNA cleavage and polyA specificity
*ima-3*	**3.8**	**102.6**	ns	ns	Importin alpha nuclear transport factor
*elt-2*	**7.5**	**>>>**	**3.2**	**5.8**	Transcription factor; intestinal
*let-70*	**9.7**	**31.6**	**4.7**	**2.9**	E2 ubiquitin conjugation enzyme
*ire-1*	**13.0**	ns	ns	ns	Kinase; ER UPR
*ufd-1*	ns	**1.7**	ns	ns	Ubiquitin fusion degradation
*cpf-2*	ns	**2.7**	ns	ns	mRNA cleavage and polyA factor
*let-92*	ns	**3.9**	**3.2**	**25.1**	Serine/threonine protein phosphatase
*arf-3*	ns	ns	**1.5**	**1.9**	ADP-ribosylation factor
*dpy-22*	ns	ns	**1.6**	ns	Transcription; mediator
*kin-1*	ns	ns	**2.0**	ns	Serine/threonine protein kinase
*sdc-2*	ns	ns	**2.5**	ns	Transcriptional repression; dosage comp.
*gob-1*	ns	ns	**3.2**	ns	Trehalose synthesis
*phi-50*	ns	ns	**5.8**	**4.3**	Mevalonate synthesis
*hda-1*	ns	ns	**5.9**	ns	Histone deacetylase
*dcp-66*	ns	ns	**13.9**	ns	Deacetylase; NuRD complex
*lin-40*	ns	ns	**17.2**	**2.2**	Deacetylase; NuRD complex
*daf-16*	ns	ns	**96.3**	ns	Transcription factor; insulin/IGF-1 sig.
*skr-1*	ns	ns	ns	**1.5**	Ubiquitin ligase complex component
*cul-1*	ns	ns	ns	**1.8**	Ubiquitin ligase complex component
*skn-1*	ns	ns	ns	**2.2**	Transcription factor; stress, detox
*nekl-2*	ns	ns	ns	**3.3**	Serine/threonine protein kinase

Expression of stress-responsive promoter::gfp fusions was quantified following treatment with an inducing toxin or genetic disruption of insulin/IGF-1 signaling. The ER UPR gene *hsp-4* is induced by the inhibitor of N-linked glycosylation tunicamycin (column 1), the Mt UPR gene *hsp-6* by the ETC complex III inhibitor antimycin (column 2), the superoxide dismutase *sod-3* by temperature sensitive inactivation of the *daf-2*(e1370) insulin/IGF-1 receptor (column 3) and the detoxification and oxidative stress response gene *gst-4* by the ETC complex IV inhibitor sodium azide (column 4). Treatments were applied to day 1 adult animals raised at 20°C. RNAi clones targeting ∼1500 candidate genes were screened for suppression of GFP expression under inducing conditions. All candidates were screened in three primary replicates of 50 animals and phenotypes were verified in five or more additional replicates. Phenotypes of 29 gene inactivations found to inhibit the induction of at least one cytoprotective pathway were quantified using the automated Molecular Devices ImageXpress Micro imaging platform. Fold decrease in fusion gene expression was quantified against vector-treated controls. Data represents the fold decrease in median expression in 4 to 8 replicates of 50 animals. For clarity, results that did not meet our threshold of significance (ns) are not shown. The symbol >>> denotes that sample fluorescence values did not differ significantly from background, precluding the quantification of fold change. We identify 15 genes required for expression of *hsp-4* following treatment with tunicamycin (column 1), 14 genes required for expression of *hsp-6* following treatment with antimycin (column 2), 14 genes required for expression of *sod-3* in a temperature shifted *daf-2*ts mutant (column 3), and 15 genes required for expression of *gst-4* following treatment with sodium azide (column 4) with significant overlap amongst functions (Figure S3). Results include well-studied canonical regulators of cytoprotective functions, including *ire-1*, *daf-16* and *skn-1*, which act specifically in the *hsp-4*, *sod-3* and *gst-4* stress response pathways, respectively. Regulation of the expression of the heat shock response p*hsp-16.2*::gfp in response to 1 hour treatment of 37°C and expression of a constitutively expressed non-stress-responsive p*sur-5*::gfp were quantified as controls (Table S4). Expression of endogenous loci was quantified by qPCR (Table S5).

The 29 regulators of cytoprotection identified are annotated to function in RNA processing (*cpsf-2*, *cpsf-4*, *cpf-2*), protein degradation (*pas-3*, *let-70*, *ufd-1*, *skr-1*, *cul-1*), deacetylation (*sdc-2*, *hda-1*, *dcp-66*, *lin-40*), phosphorylation (*wnk-1*, F18F11.5, *let-92*, *kin-1*, *nekl-2*), transcription (*mdt-26*, *dpy-22*, *elt-2*) and other activities (Table 1). Fourteen have been annotated as candidate cofactors for miRNA function, four as cofactors of RNAi and eight as positive regulators of lifespan extension in the long-lived *daf-2* mutant [59], [60], [61]. Of the eight putative insulin/IGF-1 signaling factors, five were found to potently regulate the transcription of *sod-3* downstream of *daf-2* [61].

**Figure 1:**
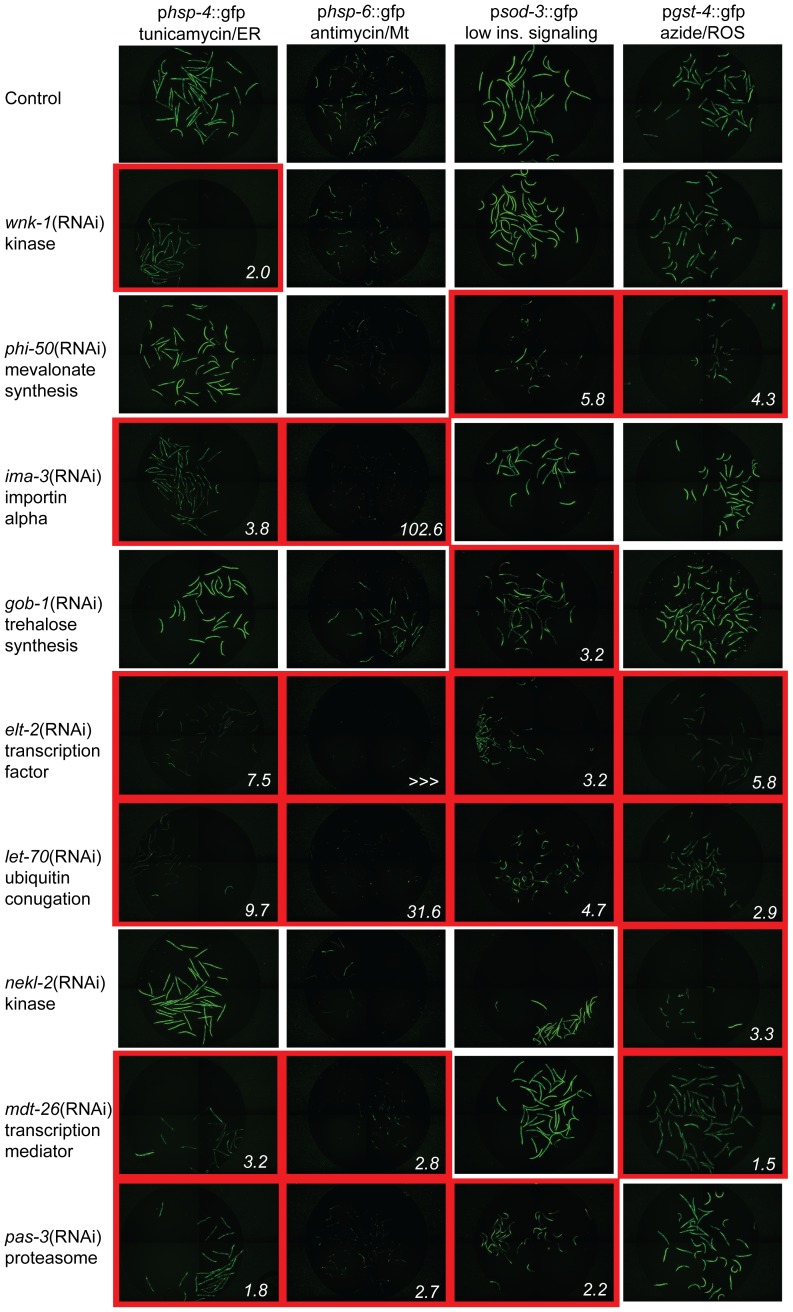
Xenobiotic response regulatory factors are specific to one or more cytoprotective pathways. Animals carrying promoter::gfp fusions to genes in key cytoprotective responses were exposed to stimuli that normally induce the expression of these constructs. These include *hsp-4*, an ER UPR gene induced by treatment with tunicamycin (column 1), *hsp-6*, an Mt UPR gene induced by treatment with antimycin (column 2), *sod-3*, an oxidative stress response gene induced in a temperature-sensitive *daf-2* mutant background (column 3), and *gst-4*, an oxidative stress response and detoxification gene induced by treatment with sodium azide (column 4). Gene inactivations found to inhibit the expression of one or more of these gfp fusions include RNAi of *wnk-1* (row 2, suppresses *hsp-4* response), *phi-50* (row 3, suppresses *sod-3* and *gst-4* responses), *ima-3* (row 4, suppresses *hsp-4* and *hsp-6* responses), *gob-1* (row 5, suppresses *sod-3* response), *elt-2* (row 6, suppresses *hsp-4*, *hsp-6*, *sod-3* and *gst-4* responses), *let-70* (row 7, suppresses *hsp-4*, *hsp-6*, *sod-3* and *gst-4* responses), *nekl-2* (row 8, suppresses gst-4 response), *mdt-26* (row 9, suppresses *hsp-4*, *hsp-6* and *gst-4* responses) and *pas-3* (row 10, suppresses *hsp-4*, *hsp-6* and *sod-3* responses). Representative images of conditions with decreased cytoprotective gene expression are outlined in red, and the fold reduction in GFP expression, quantified in Table 1, is shown in the lower right. None regulate the expression of the constitutively expressed gene fusion p*sur-5*::gfp and only *elt-2* regulates expression of the heat shock responsive gene fusion p*hsp-16.2*::gfp, suggesting specificity to stress functions (Table S4). Endogenous gene expression was measured by qPCR (Table S5).

Sixteen gene inactivations that disrupt the coupling of cellular dysfunction to cytoprotective gene activation demonstrate specificity to one of our four tested cytoprotective gene fusions, including the canonical stress response regulatory factors *daf-16*, *skn-1*, and *ire-1* (Table 1, Figure 1, Figure S3). The canonical factors score most strongly amongst these, with the exception of *nekl-2*, which regulates the expression of *gst-4*::gfp 50% more potently than *skn-1*. F53F4.11, *cpf-2* and *dcp-66* are also noteworthy as the most potent previously unidentified pathway-specific gene inactivations, regulating the expression of *hsp-4*, *hsp-6* and *sod-3* fusion genes, respectively. We observe the greatest degree of regulation, however, amongst the 16 regulators of cytoprotective gene expression that regulate 2 or more of the tested cytoprotective pathways. *lin-40* gene inactivation disrupts p*sod-3*::gfp induction 17-fold, and p*gst-4*::gfp 2-fold. *let-92* gene inactivation results in the most potent disruption of *p*gst-4::gfp induction, decreasing expression 25-fold, and inhibiting induction of p*sod-3*::gfp and p*hsp-6*::gfp by 3- and 4-fold, respectively. *ima-3* and *elt-2* gene inactivations both dramatically decrease induction of p*hsp-6*::gfp by antimycin. *ima-3* gene inactivation additionally inhibits the induction of *p*gst-4::gfp (4-fold). *elt-2*, like *let-70* and *cpsf-2*, is required for the appropriate regulation of all four tested cytoprotective pathways (Table 1, Figure 1). We conclude that the cytoprotective pathways upregulated by conditions that increase longevity are regulated by both distinct and shared genetic components (Table 1, Figure S3). We speculate that shared regulatory genes may be upstream of pathway-specific factors, though the complexity of this regulatory network remains unexplored.

### Lifespan Extension Requires Cytoprotective Gene Induction

If the cytoprotective pathways normally induced by conditions that confer increased longevity are essential for that increase, decoupling their induction might shorten the lifespan of long-lived mutants more than that of wild-type animals. To test this hypothesis, we asked whether the 29 gene inactivations that disrupt cytoprotective gene induction also abrogated the increase in lifespan conferred by mitochondrial dysfunction (*isp-1;ctb-1*), reduced feeding (*eat-2*) or disruption of insulin/IGF-1 signaling (*daf-2*).

**Figure 2:**
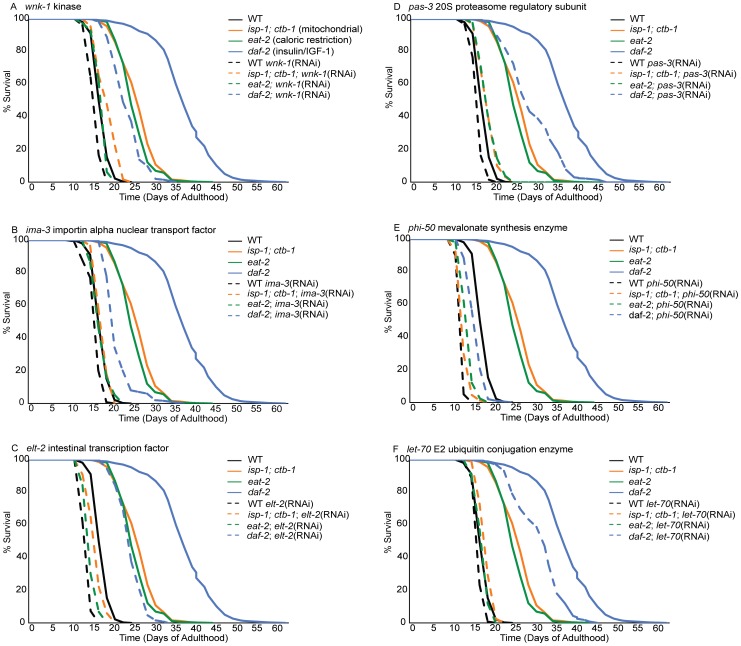
Loss of cytoprotective gene activation dramatically reduces lifespan extension in long-lived mutants but not controls. Lifespans of animals treated with empty-vector RNAi or with inactivation of *wnk-1* (A), *ima-3* (B), *elt-2* (C), *pas-3* (D), *phi-50* (E) and *let-70* (F) were determined from three replicates comprising, an average of 103 worms per condition. Lifespan was measured in wild-type N2 controls (black lines) and three long-lived mutants, including a mitochondrial mutant (*isp-1*;*ctb-1*, orange lines), an insulin/IGF-1 signaling mutant (*daf-2*, blue lines) and a feeding mutant that models caloric restriction (*eat-2*, green lines). Data for control (empty vector-fed) animals of each strain are represented by solid lines and RNAi-treated animals by dashed lines. Inactivation of these cytoprotective response regulatory genes dramatically reduces lifespan extension in at least one long-lived mutant background, but not in wild-type controls.

Inactivation of an idealized lifespan regulatory gene would reduce the lifespan of a long-lived strain to that of the control strain (N2) without perturbing wild-type lifespan. In these experiments, 12 of 29 tested gene inactivations abrogate 2/3 or more of the lifespan extension observed in *eat-2*, *isp-1* and/or *daf-2* mutants (Table 2). These gene inactivations shorten wild type lifespan much less dramatically, differentiating these lifespan-regulatory gene inactivations from generalized sickness. While *dcp-66*, *pas-3* and *arf-3* exert their largest suppression of lifespan in *isp-1*, inactivation of *cpf-2*, *wnk-1* and *nekl-2* are most potent in the *eat-2* mutant (Table 2, Figure 2). Of these, however, only *dcp-66*, which reduces lifespan extension in a mitochondrial mutant (*isp-1*) by 87%, does not significantly influence lifespan extension in at least one additional mutant. The remaining 6 gene inactivations, including *phi-50*, *ima-3*, *gob-1*, *ufd-1*, *let-70*, and *elt-2*, are critical to lifespan extension in both the *isp-1* and *eat-2* mutants (Table 2, Figure 2). Two of these, *phi-50* and *ima-3*, also reduce the lifespan of *daf-2* mutants by more than 2/3 (Table 2, Figure 2). These phenotypes represent the most potent inhibitions of lifespan extension. Applying a less conservative standard of 15% reduction in lifespan extension, 25 of 29 regulators of cytoprotective gene induction suppress the extension of lifespan in at least one long-lived strain (Table 2). Cumulatively, we find that genes required for the appropriate transcriptional response to xenobiotic stress and disruption of insulin/IGF-1 signaling contribute to diverse axes of lifespan extension, and in some cases, in particular *phi-50* and *ima-3*, to all three studied lifespan extension axes (Table 2, Figure 2).

**Table 2:** Cytoprotective gene regulation is essential to lifespan extension by diverse mechanisms.

	Control	*isp-1*; *ctb-1*	*eat-2*	*daf-2*	Control	*isp-1*; *ctb-1*	*eat-2*	*daf-2*
	Mean Lifespan (Days)				Δ Lifespan	Δ Lifespan Extension		
Control	18.1	27.0	26.2	38.7	0%	0%	0%	0%
*phi-50*	12.9	13.6	14.5	16.4	−29%	**−90%**	**−72%**	**−76%**
*ima-3*	16.9	18.5	18.1	22.2	−7%	**−82%**	**−85%**	**−72%**
*wnk-1*	16.5	19.6	18.2	24.4	−9%	**−62%**	**−78%**	**−58%**
*elt-2*	14.4	16.7	15.4	25.3	−21%	**−67%**	**−84%**	**−33%**
*pas-3*	16.8	19.3	19.5	30.2	−7%	**−70%**	**−65%**	**−30%**
*cpf-2*	17.1	21.6	19.1	30.9	−6%	**−47%**	**−74%**	**−29%**
*let-70*	17.2	19.1	18.2	32.2	−5%	**−78%**	**−87%**	**−23%**
*mdt-26*	15.7	22.5	19.6	29.4	−14%	ns	**−43%**	**−23%**
*cpsf-4*	20.8	26.9	26.3	39.1	14%	**−40%**	**−40%**	**−22%**
*arf-3*	18.1	20.8	22.7	34.5	0%	**−70%**	**−43%**	**−20%**
*gob-1*	17.3	19.3	19.4	47.2	−5%	**−76%**	**−73%**	ns
*ufd-1*	16.8	18.3	19.2	33.1	−7%	**−81%**	**−68%**	ns
*nekl-2*	13.7	17.5	15.7	33.5	−24%	**−44%**	**−67%**	ns
*let-92*	15.7	19.0	18.8	32.4	−14%	**−56%**	**−55%**	ns
*cul-1*	18.0	24.9	22.1	40.8	−1%	**−22%**	**−49%**	ns
C06A8.2	17.1	22.4	21.8	33.9	−6%	**−37%**	**−39%**	ns
*skr-1*	19.1	23.4	25.1	38.1	5%	**−54%**	**−29%**	ns
*sdc-2*	15.5	18.6	20.7	35.7	−14%	**−59%**	**−25%**	ns
Y50D7A.11	19.0	27.8	26.2	37.8	5%	ns	**−16%**	ns
*kin-1*	13.3	17.1	18.4	36.4	−27%	**−43%**	**−15%**	ns
*dcp-66*	12.7	13.5	17.5	34.2	−30%	**−87%**	ns	ns
*hda-1*	16.2	20.1	24.2	37.8	−11%	**−51%**	ns	ns
*lin-40*	17.3	23.1	25.6	38.7	−5%	**−31%**	ns	ns
*dpy-22*	17.6	24.6	25.9	41.9	−3%	**−18%**	ns	ns
F53F4.11	19.4	27.5	29.4	38.7	7%	**−15%**	ns	ns
*sptl-1*	18.6	26.6	27.6	37.9	2%	ns	ns	ns
*cpsf-2*	18.9	30.0	29.4	39.6	4%	ns	ns	ns
F18F11.5	17.6	29.6	26.9	43.3	−3%	ns	ns	ns
*rab-10*	18.3	28.6	26.1	39.6	1%	ns	ns	ns

Twenty-nine gene inactivations required for the appropriate induction of cytoprotective responses following stress (Table 1) were screened for lifespan phenotypes. Each gene inactivation, or an empty-vector RNAi control, was fed to wild-type (N2) animals and each of three long-lived mutants, including the *isp-1;ctb-1* mitochondrial mutant, *daf-2* insulin/IGF-1 signaling mutant and *eat-2* feeding-defective mutant. Lifespan was measured in three replicates comprising an average of 103 worms per condition. Data is presented as mean lifespan (columns 1–4), change mean lifespan in comparison to an empty vector RNAi control (column 5), or as the change in the extension of lifespan induced by each long-lived mutant (columns 6–8; for clarity, only decreases >15% are shown). Only 5 of the 29 gene inactivations result in a 15% or greater reduction in wild-type (N2) lifespan, and most (20 of 29) reduce wild-type lifespan by less than 10% (column 5). Of the 29 genes, 25 demonstrate a significantly greater effect in at least one long-lived mutant background, and 16 gene inactivations abrogate 50% or more of the lifespan extension normally induced by at least one of the tested mutant backgrounds. Because these gene inactivations have small effects on wild-type lifespan but large effects on lifespan extension in long-lived mutant backgrounds, we propose that these gene inactivations are not simply progeric but instead play specific roles in lifespan extension.

Many gene inactivations that inhibit lifespan extension also decrease stress tolerance. To reveal the role of cytoprotective response regulatory genes in the tolerance of xenobiotic stress, we inactivated the 29 genes identified in the screen and challenged animals with sublethal (LD30) doses of antimycin, sodium azide, cadmium chloride and paraquat. These toxins parallel the conditions utilized in the screen. Antimycin targets the function of mitochondrial ETC complex III and activates p*hsp-6*::gfp. Sodium azide disrupts the final step of electron transport, blocking energy production and releasing reactive oxygen species, leading to the induction of p*gst-4*::gfp [58]. Cadmium, like tunicamycin, induces the ER stress response and cadmium tolerance is dependent upon a functional ER UPR [62]. Paraquat survival has been utilized as a measure of ROS tolerance, known to result from the activation of cytoprotective genes downstream of insulin/IGF-1 signaling, such as the superoxide dismutase *sod-3* [53].

**Figure 3:**
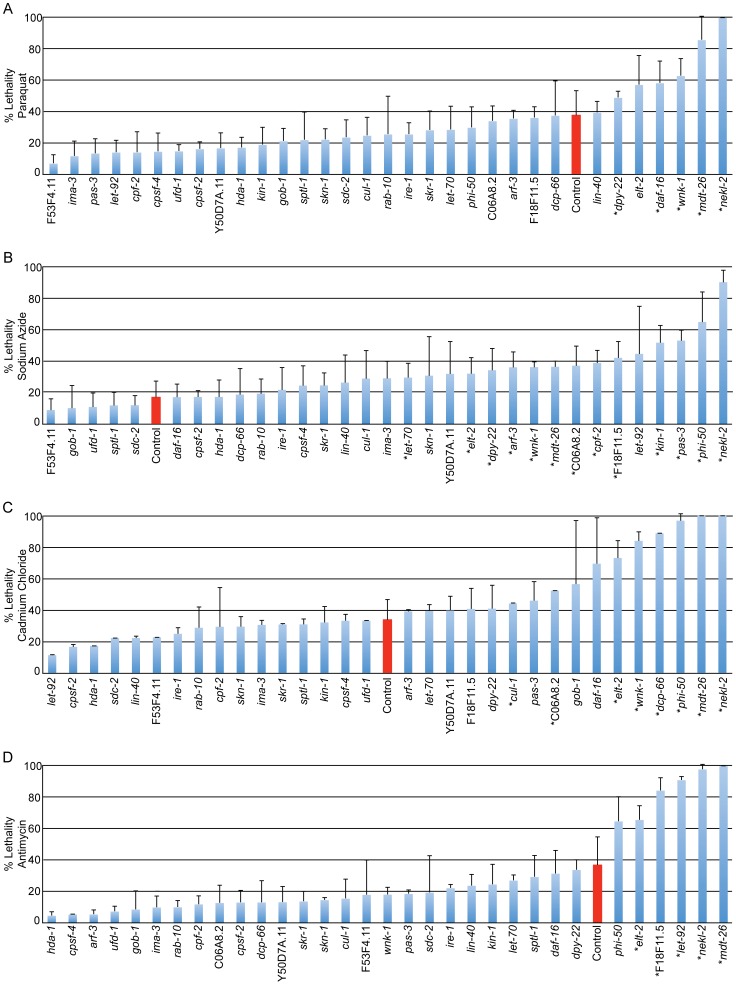
Regulation of cytoprotective gene activation is required for xenobiotic stress tolerance. Genes found to regulate cytoprotective responses in our screen were inactivated in *rrf-3*ts sterile mutant animals. Animals were raised to day 3 adulthood on solid media and transferred to solution containing sublethal (∼LD30) treatments of 24 mg/ml paraquat (A), 22 µg/ml sodium azide (B), 5.2 mg/ml cadmium chloride (C), 696 µg/ml antimycin (D) or solvent alone (Figure S4). Animals were incubated in solution for 18 hours and survival was measured by spontaneous movement. An average of 94 animals were scored for each condition. Control samples treated with empty vector (L4440) RNAi are shown in red, with all other samples treated with gene inactivations that disrupt cytoprotective responses. Gene inactivations are ordered by decreasing survival within each panel. Phenotypes demonstrated by inactivation of *nekl-2*, *mdt-26*, *wnk-1*, *phi-50* and *elt-2* are amongst the most robust. Gene inactivations do not reduce survival in the presence of solvent alone (Figure S4). Error bars display S.D., asterisks indicate significantly decreased survival, p<0.05.

Inactivation of 16 of 29 genes that disrupt induction of the cytoprotective GFP fusion genes, also disrupted the ability of animals to survive exposure to xenobiotics (Figure 3). Eleven of the sixteen xenobiotic-sensitive gene inactivations (*phi-50*, *wnk-1*, *nekl-*2, *mdt-26*, *let-70*, *arf-3*, *elt-2*, *dpy-22*, *let-92*, F18F11.5, and C06A8.2) enhance sensitivity to the xenobiotic that pairs with the compromised cytoprotective response. The strongest examples of this correlation include *phi-50* and *nekl-2* (*pgst-4::gfp/sodium* azide), *elt-2*, *wnk-1* and *mdt-26* (p*hsp-4*::gfp/cadmium chloride), *let-92*, *elt-2* and *mdt-26* (p*hsp-6*::gfp/antimycin) and *dpy-22* (p*sod-3*::gfp/sodium azide). Of the eleven total gene inactivations that pair in this way, seven are also susceptible to additional xenobiotics, suggesting that the pathways examined serve cytoprotection more extensively than previously predicted. In addition, five gene inactivations (*dcp-66*, *pas-3*, *kin-1*, *cpf-2* and *cul-1*) are sensitive only to xenobiotics that do not directly pair with the observed deficit in cytoprotective gene induction, further demonstrating the complexity of stress responsive gene networks and their protective functions. None of the 29 gene inactivations significantly decreased survival following treatment with drug solvent controls alone (Figure S4).

Cumulatively, *phi-50*, *ima-3*, *elt-2*, *nekl-2*, *wnk-1*, *let-92*, *mdt-26*, and *let-70* stand out amongst the 29 genes that disrupt cytoprotective response (Table S6). *wnk-1*, *phi-50* and *elt-2* are severely sensitive to multiple xenobiotic stress conditions, but not control conditions, and modulate lifespan extension in all three tested axes (insulin/IGF-1 signaling, mitochondrial function and caloric restriction). While *ima-3* and *let-92* do not demonstrate xenobiotic sensitivity under the tested conditions, and *let-70* only a subtle sensitivity to sodium azide, they are amongst the most robust suppressors of cytoprotective transcription and suppress lifespan extension in all three long-lived mutants. *mdt-26* and *nekl-2* are sensitive to all four xenobiotic treatments and suppress lifespan extension in two of the three long-lived mutants tested (*daf-2* and *eat-2* or *isp-1*;*ctb-1* and *eat-2*, respectively). In total, we identify 15 regulators of cytoprotection that are required for tolerance of xenobiotic stress and lifespan extension (Table S6).

## Discussion

Stress tolerance and lifespan extension are remarkably correlated. The contradictory extension of lifespan by ostensibly deleterious conditions, and the concomitant induction of stress tolerance, suggests that lifespan extension may occur through the hormetic induction of damage-buffering cytoprotective mechanisms. We have identified the cytoprotective pathways that are upregulated by conditions that extend lifespan. In a screen of 160 gene inactivations that increase lifespan, the most potent lifespan extension phenotypes were defined by the induction of suites of cytoprotective genes (Figure S2) [32], [35], [63]. To identify upstream regulatory genes in xenobiotic responses, we designed an RNAi screen to detect gene inactivations that disrupt the normal induction of p*hsp-6*::gfp, p*gst-4*::gfp and p*hsp-4*::gfp by toxins, and the activation of p*sod-3*::gfp by low insulin/IGF-1 signaling. The induction of cytoprotective longevity-modulatory pathways by toxins may be the normal biological context in which these pathways function, having evolved as countermeasures to the xenobiotic and environmental challenges that animals encounter. Because xenobiotics and lifespan extending gene inactivations engage the same cytoprotective, physiological and behavioral responses, xenobiotic responses may be triggered by direct surveillance of cell functions, which would provide broad, adaptive utility in toxin detection [64], [65], [66].

We identified 29 gene inactivations that decouple normal transcriptional responses to toxins and environmental stress. While some gene inactivations were specific to one toxic modality, such as mitochondrial dysfunction, others affected multiple, distinct toxin response pathways. The identified genes may act in damage surveillance, signaling, or the transcriptional coordination of cytoprotective responses by acting either within cells and tissues or across tissues by an as-yet-undefined endocrine mechanism. Gene inactivations specific to one toxin may act in dedicated surveillance pathways, while those that affect multiple, distinct responses may identify points of signal convergence.

Many of the gene inactivations that disrupt the coupling of cellular dysfunction and the transcription of cytoprotective responses in the screen are annotated phosphorylation or transcription factors. The kinase *nekl-2*, which we identify in the regulation of *gst-4* expression, is necessary for the nuclear localization of *skn-1* following oxidative stress [67]. The kinase *wnk-1*, which we identify as a regulator of the ER stress response, has previously been placed upstream of effector genes in the osmotic stress response [68]. *let-92*, the catalytic subunit of protein phosphatase 2A, stands out as a potent regulator of *gst-4* expression, suggesting it may play a critical role in the regulation of *skn-1* activity. The transcription factor *elt-2* is expressed exclusively in the intestine, a critical tissue in xenobiotic detection and detoxification in *C. elegans*. Targets of *elt-2* include osmoprotective and innate immune responses, detoxification and oxidative defenses and metal detoxification, as well as the transcription factor *pha-4* and, potentially, *skn-1*. This multitude of key cytoprotective functions is consistent with our finding that *elt-2* is required for appropriate expression of *hsp-6*, *hsp-4*, *sod-3* and *gst-4*.

We identified five components of the ubiquitin proteasome system (*pas-3*, *let-70*, *ufd-1*, *skr-1*, *cul-1*). Proteasome regulatory components like *aip-1*, and potentially *skn-1*, are believed to prolong lifespan by stimulating the degradation of damaged proteins [69]. Others, such as the E3 ubiquitin ligase *vhl-1*, serve regulatory functions by degrading key signaling components [70]. The E2 ubiquitin conjugation factor *let-70* regulates all four tested cytoprotective responses (p*hsp-6*::gfp, p*hsp-4*::gfp, p*sod-3*::gfp, p*gst-4*::gfp), but not the heat shock response (p*hsp-16.2::gfp*) or a constitutively expressed control (p*sur-5*::gfp). It has been previously shown that let-70 interacts with numerous chaperones, contributes to the DNA damage response, and that inactivation of *let-70* increases the size of aggregates in a polyglutamine model of protein aggregation [71]. The diverse functions of *let-70*, one of 22 E2 ubiquitin conjugation factors in *C. elegans*, are consistent with its interaction with a much larger pool of target-specifying ubiquitin-protein ligases (E3s).

We identify three deacetylases (*hda-1*, *dcp-66*, *lin-40*), all of which regulate the expression of p*sod-3*::gfp downstream of insulin/IGF-1 signaling. Studies of chromatin modification have demonstrated that both silencing and desilencing epigenetic marks regulate lifespan [72], [73].

Other genes of interest include *ima-3* and *phi-50* (Table 1, Table 2). *ima-3*, one of three importin alphas that channel NLS-tagged molecules into the nucleus, stands out as the most potent regulator of the mitochondrial UPR we identify. Our results suggest that *ima-3* may participate in the transport of stress regulatory factors into the nucleus. *phi-50*, which regulates p*sod-3*::gfp and p*gst-4*::gfp, is orthologous to the human hydroxymethylglutaryl-CoA synthases 1 and 2 (HMGCS1, 2) [74]. HMGCS1 is required for the prenylation of proteins such as GTPases, which are essential to organelle homeostasis and many signaling cascades, while HMGCS2 mediates the response to fasting [75], [76].

Eighteen genes found to disrupt the induction of cytoprotective responses serve putative microRNA or RNAi functions, suggesting that small RNAs regulate stress response. As most microRNAs in *C. elegans* are not individually essential for viability under standard laboratory conditions, the possibility that they fulfill conditional functions such as stress response is particularly appealing [77]. Small RNAs are attractive candidates for stress response regulation, as they are rapidly inducible and the suppression of protein levels they mediate is rapidly reversible [78], [79]. Small RNAs may be generated without translation and spread easily amongst cellular compartments or from cell to cell [79], [80]. Roles for small RNAs in stress responsive gene regulation are emerging in bacterial, plant and mammalian systems [81], [82], [83], [84], [85], [86], [87]. The capacity of small RNA pathways to mediate the expression of duplicated and genetically linked genes may contribute to their potential to act as key regulators of xenobiotic response, as detoxification genes are known to form long tandem arrays by duplication [88]. Like canonical stress response regulatory genes, some small RNAs have been found to regulate longevity [89]. Regulation of cytoprotective genes by siRNA- or miRNA-mediated silencing is likely indirect under the tested conditions because inactivation of genes required for silencing would be expected to increase, not decrease, the expression of a direct target. Additionally, the transgenes utilized were promoter fusions.

The complexity of stress response is increasingly evident, challenging the presumption that cytoprotective pathways are genetically independent. Translation initiation factor 2 (eIF2α) integrates signals from four stress-activated kinases, each responding to diverse stress stimuli including oxidative damage, amino acid starvation, infection and ER stress. Another example is found in the transcription factor *slr-2*, which co-regulates a suite of diverse stress response genes, including *hsp-16.2* (heat shock), *hif-1* (oxidative stress) and *gpdh-1* [90]. The insulin/IGF-1 signaling pathway contributes to the tolerance of heat, radiation, osmotic stress, oxidative damage and heavy metals, as well as pathogens. It is not surprising that some of the genes identified in our study engage more than one cytoprotective mechanism.

The regulators of cytoprotection we identified contribute to lifespan extension in three distinct long-lived mutants: *isp-1* (mitochondrial function), *eat-2* (caloric intake) and *daf-2* (insulin/IGF-1 signaling). Nearly all of the identified regulators of cytoprotection contributed to lifespan extension in at least one of these mutants and many modulate lifespan extension in at least two conditions. Because previously identified positive regulators of lifespan, such as *daf-16* and *hsf-1*, manifest the cumulative benefit of large suites of co-regulated genes, we suggest that the stress response and lifespan regulatory genes identified here similarly abrogate the induction of many downstream cytoprotective effectors. Our finding that nearly all gene inactivations that disrupt the induction of cytoprotective pathways by toxins also disrupt longevity extension suggests a tight coupling of these pathways. Increased longevity may be the cumulative result of cytoprotective pathway induction or, alternatively, a coregulated output of xenobiotic response analogous to the hormonal pathways of lifespan regulation engaged by insulin/IGF-1 signaling mutants. Our results suggest that xenobiotic and environmental response mechanisms underpin diverse models of longevity extension, with the potential to unify the study of long-lived animals.

Lifespan poses an evolutionary conundrum, as the genetic determination of lifespan ostensibly suggests post-reproductive selection. Our data suggests that lifespan-determining genes do not specify lifespan *per se*, but rather the activity of damage-buffering cytoprotective pathways normally engaged only in response to stress stimuli, such as toxins. Cytoprotective programs must be subject to Darwinian evolution, selected pre-reproductively to maintain the viability of larval and young adult animals in the presence of xenobiotic and environmental challenges. Post-reproductive adults could engage the same programs. The functions of these pathways are expected to be highly regulated, since they marshal essential resources, such as iron for cytochrome p450s or ATP for chaperones and transporters, away from anabolic pathways and reproduction; organisms that upregulate these pathways continuously would be outcompeted by those who regulate them conditionally [91].

We have identified upstream regulatory components of longevity and xenobiotic response pathways, the overlap of which supports our hypothesis that longevity pathways evolved as xenobiotic and environmental stress response programs. Our results reveal the complex networking of cytoprotective gene regulation. The genes we have identified may act in the detection of stress stimuli, the transduction of a resulting signal or the direct regulation of the transcription of stress response effectors. We find that these upstream regulators play central roles in both xenobiotic stress tolerance and the extension of lifespan in several canonical long-lived strains, including *eat-2*, *isp-1* and *daf-2*. Xenobiotic and environmental stress response pathways may underpin many current models of longevity extension. The xenobiotic hypothesis of aging invokes hormesis, a phenomenon observed from microorganisms to humans, highlighting the possibility of a xenobiotic approach to longevity extension in humans.

## Materials and Methods

### Strains

Fluorescent strains p*hsp-6*::gfp(sj4100), p*hsp-60*::gfp(sj4058), p*hsp-4*::gfp(sj4005), p*sod-3*::gfp(cf1553), p*gst-4*::gfp(cl2166)., p*hsp-16.2*::gfp(tj375) and p*fat-7*::gfp(bc15777) were obtained from the *C. elegans* genome center (CGC). p*gpdh-1*::gfp(vp198) was obtained courtesy of Kevin Strange. Bristol(N2), *rrf-3*(pk1426) and *daf-16*::gfp(gr1352) were obtained from the Ruvkun laboratory. pF55G11.7::gfp(hd92), p*lys-1*::gfp(hd102), p*lys-7*::gfp(hd100) and p*nlp-29*::gfp(hd101) were obtained courtesy of Scott Alper. Long lived strains were *daf-2*(e1370), *eat-2*(ad465) and *isp-1*;*ctb-1*(mq989).

### Activation of GFP Fusion Genes by RNAi

RNAi clones were grown overnight in LB with 100 µg/ml ampicillin and seeded 100 µl/well to 24-well 5 mM IPTG worm plates. Clones were induced overnight at room temperature. Synchronized L1 worms were raised on RNAi at 20°C, 50 animals/well. Fluorescence was assayed at 48, 72 and 96 hours. Scores were recorded from 0 (no expression) to 4 (strong expression). For lethal clones, worms were grown to young adulthood at 20°C on empty-vector RNAi (L4440) before treatment, with subsequent scoring after 24, 48 and 72 hours. Each clone was scored in three trials. Resulting data was clustered using the open source software Cluster 3.0 with hierarchical uncentered correlation of average linkage and visualized using Java TreeView.

### Activation of GFP Fusion Genes by Stress

RNAi clones were cultured overnight in LB with 100 µg/ml ampicillin and seeded 100 µl/well to 24-well 5 mM IPTG worm plates, each well containing 1.5 mL agar. Synchronized L1 transgenic strains (SJ4100, sj4005, cf1553;e1370, cl2166) were distributed to RNAi, 50 animals/well, and raised to young adulthood (56 hours) at 20°C. At this time, each well was treated with 0.5 µl 20 mg/ml antimycin in EtOH (sj4100), 1.8 µl10 mg/ml tunicamycin in DMSO (sj4005), 17 µl1 mg/ml sodium azide in water (cl2166) or 1 h 37°C heat shock (tj375). Toxins were diluted in water to 20 µl/well and applied directly to the agar wells. Expression was assayed after 8 hours (tunicamycin, heat), or 24 hours (antimycin, sodium azide). For *sod-3*(cf1553), p*sod-3*::gfp;*daf-2*(e1370)ts was raised to young adulthood at 15°C and shifted to 25°C with GFP expression assayed after 12 hours. All clones in the primary screen were scored in three replicates. Candidate stress response regulatory genes were subsequently verified in five or more additional replicates.

### Quantification of Fluorescence

Treated worms (see activation of fusion genes by stress in materials and methods) were washed into 200 µl M9 containing 0.3 mg/ml lavamisole and 0.005% Triton X-100, and concentrated in a 96-well pate by centrifugation for 1 minute at 500 RPM, then transferred to 96-well glass slides with a final liquid volume of 5 µl/well. Imaging of slides was automated using Molecular Devices ImageXpress Micro imaging platform with MetaXpress software. Device captures four images per well, which are tiled to construct full wells. Images are captured in both in both GFP and bright field channels. Custom MATLAB (The Mathworks, Natick, MA) scripts distinguish well boundaries by blurring the image and applying a threshold of L*F where L is determined by Otsu's method and F = 0.9. To identify worms, bright field images are bottom hat filtered to decrease variability in background intensity. Otsu's method and a size filter are applied to distinguish objects from background and debris. An outlier method is applied in place of Otsu's when effectiveness is low (<0.7). Fluorescence of worm objects is averaged from the median intensity of each well after background subtraction. Results were averaged from four to eight replicates for each experimental condition and the p*sur-5*::gfp control, with two replicates for the p*hsp-16.2*::gfp control. Significance was determined by a one-tailed t-test, p = 0.05, and fold decreased expression >1.5× without multiple tests correction. Venn diagrams were generated using the online BioInfoRx Venn diagram tool available at <bioinforx.com/free/bxarrays/venndiagram.php>.

### Quantification of Endogenous Gene Expression by qPCR

Wild-type N2 animals were raised on HT115 bacteria on 10 cm agarose plates at 20°C and treated with cytoprotective response-inducing toxins (see activation of fusion genes by stress in materials and methods). Animals were harvested after 8 hours (antimycin, tunicamycin) or 24 hours (sodium azide). In the case of *sod-3*, e1370 animals were raised to young adulthood at 15°C and shifted to 25°C and harvested after 12 hours. To isolate total RNA, animals were washed, resuspended in trizol, frozen and homogenized by grinding. RNA was isolated by chloroform extraction followed by ethanol precipitation. Reverse transcription was carried out with the Ambion AM1710 Retroscript RT-PCR kit. Quantitative PCR reactions utilized 12.5 µl Bio-Rad iQ SYBR Green PCR Supermix (170-8880) with 2 µl template, 5.5 µl water and 5 µl each of two of the following paired oligos: *hsp-4* GAGAACACAATTTTCGACGCC/GACTTGTCGACGATCTTGAACGG; *hsp-6* GATAAGATCATCGCTGTCTACG/GTGATCGAAGTCTTCTCCTCCG; *sod-3* CACTATTAAGCGCGACTTCGG/CAATATCCCAACCATCCCCAG; *gst-4* GCCAATCCGTATCATGTTTGC/CAAATGGAGTCGTTGGCTTCAG. Fold change was measured in comparison to Y45F10D.4 using oligos GTCGCTTCAAATCAGTTCAGC/GTTCTTGTCAAGTGATCCGACA as described by Hoogewijs et al. 2008 [92]. Quantitative RT-PCR was carried out using a Bio-Rad C1000-CFX96RT thermocycler (3 m 95°C; 44 cycles of 95°C 10 s, 60°C 30 s, 72°C 30 s; 5 m 72°C). Experiments were carried out with four replicates of approximately 4,000 animals per replicate.

### Lifespan Analysis

Long-lived strains *daf-2*(e1370), *eat-2*(ad465) and *isp-1*;*ctb-1*(mq989) were raised to young adulthood at 20°C on 10 cm worm plates with HT115 *E. coli*. RNAi clones were cultured overnight in LB with 100 µg/ml ampicillin, seeded 400 µl/well to 6-well 5 mM IPTG worm plates and induced overnight. The young adult animals were transferred to the prepared IPTG worm plates, 40 animals/well, and FuDR was immediately applied to the plate to a final concentration of 80 µg/ml agar. On day 4 adulthood, wells were supplemented with 400 µl of additional bacteria concentrated to 10× in 5 mM IPTG M9 with 100 mg/ml ampicillin and induced for 2 hours at room temperature before application to worm plates. Lifespan was scored by touch response on alternate days with censoring. Survival statistics were calculated using SPSS Kaplan-Meier. All analyses are based upon mean lifespan. Experiments were performed with three replicates per condition and an average of 103 worms scored per condition. Significance was held to p = 0.05 within each tested strain; significance of differences in lifespan extension are based upon a threshold of 15% decrease. The DNA and RNA synthesis inhibitor 5-fluoro-2′-deoxyuridine (FUdR) was used to inhibit progeny production. Although the use of FUdR is well established and does not affect the lifespan of wild-type animals, FUdR can affect lifespan of particular mutants [93], [94], [95], [96]. For example, mutation of *tub-1* or *gas-1* extends lifespan in the presence of FUdR, but not in its absence [93], [97]. Our experiments included FUdR in both control and experimental trials, so that any FUdR effects on lifespan were controlled.

### Stress Tolerance

L1 *rrf-3*(pk1426)ts worms were synchronized overnight in M9 and raised to young adulthood at 25°C on 10 cm worm plates with HT115 *E. coli*. RNAi clones were cultured overnight in LB with 100 µg/ml ampicillin, seeded to 6-well 5 mM IPTG worm plates and induced overnight. Young adult *rrf-3* worms were distributed to RNAi, 50 animals/well, and raised for 3 days at 25°C. Worms were transferred to wells of M9 containing 24 mg/ml paraquat, 5.2 mg/ml cadmium chloride, 22 µg/ml sodium azide, or 696 µg/ml antimycin, with a total volume of 230 µl per well in a 24-well format. Animals were incubated in solution for 16 hours. Survival was then analyzed by scoring for spontaneous movement. Experiments were conducted with three replicates and an average of 94 animals scored per condition. Significance of proportion survival was determined by a one-tailed t-test, p = 0.05 without multiple tests correction. Error bars display S.D.

## Supporting Information

Figure S1 Overview of experimental approach. The experimental strategy employed in the described experiments is broken down into three steps. First, we used a collection of GFP fusions to genes known to be induced by a variety of diverse stressors to ask if they were also induced by gene inactivations that extend longevity. Clustering of the resulting data shows that patterns of stress-responsive gene activation are specific to individual paradigms of longevity extension, such as in the inhibition of metabolism or the disruption of translation. Cytoprotective responses activated in long-lived animals were found to include *hsp-6* (Mt UPR), *hsp-4* (ER UPR), *sod-3* (ROS response) and *gst-4* (detoxification and ROS response). In the second step, we used an RNAi screen to explore how these responses were regulated. Toxins and heat were used to induce their expression, and gene inactivations that disrupted the induction of these cytoprotective responses were identified. Finally, because stress response and longevity are tightly correlated, we asked whether these gene inactivations were required for the ability to tolerate lethal doses of toxins or for the extension of lifespan induced by disruption of metabolism, feeding or insulin/IGF-1 signaling, respectively, in the *isp-1*;*ctb-1*, *eat-2* and *daf-2* mutant backgrounds. We find that almost all of the new regulators of cytoprotection identified in the second step of the experimental strategy did in fact regulate stress tolerance and lifespan extension.
(EPS)

Figure S2 Expression of cytoprotective genes is a shared characteristic of long-lived animals and distinguishes functional classes. Fluorescently tagged cytoprotective genes are induced by gene inactivations that confer lifespan extension. Fusions of cytoprotective genes, as show across the x-axis, include the mitochondrial UPR effectors p*hsp-6*::gfp and p*hsp-60*::gfp, the ER UPR chaperone p*hsp-4*::gfp, the detoxification and oxidative stress responsive p*gst-4*::gfp, the oxidative stress response fusion gene p*sod-3*::gfp, an antimicrobial effector pF55G11.7::gfp and the osmotic stress response factor p*gpdh-1*::gfp. These fluorescent reporters were treated with 160 gene inactivations (Table S2) known to extend lifespan and encompassing much of the diversity of lifespan extension mechanisms, including the disruption of metabolism, translation and insulin/IGF-1 signaling(Table S2). Expression of the stress-responsive GFP fusion genes was induced by 88 of these lifespan-extending gene inactivations, shown along the y-axis (72 others not shown). Expression of cytoprotective genes is mechanistically relevant; clusters dominated by metabolism and translation are most evident. Genes with annotated functions in metabolism activate p*hsp-6*::gfp, p*hsp-60*::gfp, p*gst-4*::gfp, p*sod-3*::gfp and pF55G11.7::gfp. This group forms a core set of 25 longevity-inducing gene inactivations (22 with metabolic annotations) and six additionally defined by expression of p*hsp-6*::gfp, comprising 28 of 35 lifespan-extending gene inactivations with metabolic annotations. Another cluster expressing p*gst-4*::gfp, p*sod-3*::gfp, pF55G11.7::gfp and p*gpdh-1*::gfp comprises 8 of 9 gene inactivations within the set of 160 with annotated functions in translation. These clusters suggest functions for previously unclassified lifespan extending gene inactivations; *ril-1*, *ril-2* and T21B10.1, a homolog of mitochondrial ribosomal protein L50, cluster with gene inactivations that disrupt mitochondrial function, while ZK1127.5 and F19B6.1 cluster with those that disrupt translation. ZK1127.5 encodes an RNA cyclase and F19B6.1 encodes a nucleotide modification enzyme, consistent with a role in translation. The activation of *sod-3*, *gst-4* and F55G11.7 may comprise a core set of longevity-correlated stress responses. Activation of stress-responsive pathways not studied in this analysis may elaborate mechanisms of lifespan extension not categorized by the present analysis. Only two gene inactivations (*sams-1* and *ero-1*) engage the ER UPR chaperone *hsp-4*, suggesting that these may modulate longevity in a distinct manner, while gene inactivations that strongly activate only *gst-4* (*cct-4*, *cct-6*, *htp-3*, *sem-5*, *sas-5*, *erm-1*, *iff-1*, Y54E10BR.4, D2030.9) may act exclusively through *skn-1*.
(EPS)

Figure S3 Overlap of regulatory functions across diverse cytoprotective pathways. While canonical regulators of cytoprotective function, including *skn-1*, *daf-16* and *ire-1* and *hsf-1* demonstrate specificity to their known downstream targets, *gst-4*, *sod-3*, *hsp-4* and *hsp-16.2* respectively, many of the new regulators of cytoprotection identified in our screen regulate more than one cytoprotective pathway. While 16 of the genes identified, like *daf-16* and other canonical stress response regulatory genes, are specific to a single effector, the remaining 13 function across multiple tested stress response effectors (Table 1). Venn diagrams that detail the regulatory elements identified for *gst-4*, *hsp-4* and *hsp-6* (A); *sod-3*, *hsp-4* and *hsp-6* (B); *gst-4*, *sod-3*, and *hsp-4* (C); *gst-4*, *sod-3*, and *hsp-6* (D), suggesting elements of both specificity and networking amongst cytoprotective regulatory pathways. The greatest overlap is observed between *hsp-4* and *hsp-6*. Diagrams do not account for phenotypic strength. Results suggest the existence of a stress response regulatory network that could not be detected by studies focusing on a single cytoprotective function.
(EPS)

Figure S4 Water and ethanol controls for xenobiotic survival. Inactivation of cytoprotective response regulatory genes found to modulate tolerance of xenobiotics, including paraquat, sodium azide, cadmium chloride and antimycin (Figure 3). In these experiments, day three adult *rrf-3* animals were transferred to M9 and treated with xenobiotics solubilized in water (paraquat, sodium azide, cadmium chloride) or ethanol (antimycin). In order to ascertain whether the addition of the water or ethanol solvents to the M9 solution contributed to survival, animals were incubated in 200 µl M9 treated with water (30 ul) or ethanol (8 µl ethanol with 22 µl water) alone. This control reveals that neither the addition of water nor ethanol results in significant mortality in control (L4440) or RNAi-treated animals; survival phenotypes may be attributed specifically to the effects of the toxins. Significance was determined with a threshold of p = 0.05.
(EPS)

Table S1 Thirteen stress-responsive GFP fusion genes. We analyzed the induction of thirteen stress-responsive GFP fusion genes by gene inactivations that extend longevity. The list encompasses a variety of diverse cytoprotective functions, including unfolded protein responses, innate immunity, oxidative stress response, insulin/IGF-1 signaling and detoxification. This collection of genes and functions represents a useful but circumscribed survey of cytoprotective pathways. *Lamitina T, Huang CG, Strange K (2006) Genome-wide RNAi screening identifies protein damage as a regulator of osmoprotective gene expression. Proc Natl Acad Sci U S A 103: 12173–12178.
(DOCX)

Table S2 160 gene-inactivations known to extend longevity. We analyzed the induction of cytoprotective responses by 160 gene inactivations previously found to extend longevity in high-throughput RNAi screens (Hamilton et al. 2005, Hansen et al. 2005 and Curran and Ruvkun 2007). While not comprehensive, these high-throughput screens are the most comprehensive efforts to globally identify such genes. This circumscribed collection is appropriate to our preliminary experiments because it provides a broad canvas of many gene functions that influence longevity, including insulin/IGF-1 signaling, metabolism and translation. Lifespan extension percentages are compiled from the annotated publications, with the larger percentage lifespan extension shown if the gene is found in more than one publication. 1, Curran and Ruvkun 2007; 2, Hansen et al. 2005; 3, Hamilton et al. 2005.
(DOCX)

Table S3 Cytoprotective response regulatory genes not discussed within the manuscript. Our primary screen identified 73 candidate stress response suppressors, of which 32 were subsequently quantified and analyzed for secondary phenotypes. Forty-one gene inactivations, however, were not subjected to secondary analysis based upon phenotypic strength or functional annotations suggesting that the gene inactivation resulted in a general disruption of transcription or translation. Data reflects the results of our primary stress response suppression screen. Animals carrying the stress-responsive gene fusions p*hsp-4*::gfp, p*hsp-6*::gfp, p*sod-3*::gfp or p*gst-4*::gfp were raised to young adulthood and treated with tunicamycin, antimycin, a temperature shift in a *daf-2*ts mutant background or sodium azide, respectively, as described in methods. Results were scored visually and the fluorescence of each condition was graded on a scale of 0, meaning no decrease in fluorescence, to −4, total loss of fluorescence. Data represents the average of five or more replicates of fifty animals.
(DOCX)

Table S4 Quantification of p*hsp-16.2*::gfp and p*sur-5*::gfp fluorescence. Expression of p*hsp-16.2*::gfp and p*sur-5*::gfp was quantified following gene inactivations that disrupt stress-responsive gene induction. Data control for the possibility that these gene inactivations are not specific to cytoprotective functions and instead function generally in gene expression or transgene silencing. Expression of p*hsp-16.2*::gfp was induced by treatment at 37°C for 1 hour. Inactivation of *hsf-1*, the known master regulator of heat shock response genes, including *hsp-16.2*, reduces expression of *phsp-16.*2::gfp by 10.8 fold (data not shown). Of 29 gene inactivations found to disrupt the induction of cytoprotective genes, 27 did not significantly effect the expression of p*hsp-16.2*::gfp and two, *elt-2* and *sptl-1*, were found to contribute partially to this response. We also measured the expression of a non-stress-induced, constitutively expressed gene fusion, p*sur-5*::gfp, and found that that none of the tested gene inactivations significantly regulate this control. As a result, we conclude that none of the gene inactivations tested contribute to transgene silencing or abrogate gene induction. Significance was determined with a threshold of p = 0.05.
(DOCX)

Table S5 qPCR results. Expression of endogenous stress-responsive loci was measured by qPCR to verify phenotypes observed using fluorescent fusion genes. Our primary screen identified gene inactivations required for the induction of p*hsp-4*::gfp, p*hsp-6*::gfp, p*sod-3*::gfp and p*gst-4*::gfp upon treatment with inducing stimuli. Wild-type (N2) animals with no transgenes were raised and treated under the conditions of our primary screen (see methods) and animals were harvested from each condition with 4 replicates of approximately 4,000 worms per replicate. Expression of cytoprotective genes was quantified for animals fed an empty-vector (L4440) RNAi control or gene inactivations identified in the stress response suppression screen. Inducing conditions were applied to both vector-fed and RNAi-fed animals. GFP data represents the fold decrease fluorescence as compared to the empty-vector control and is identical to data from Table 1. qPCR data are presented as the difference in fold induction between vector and RNAi treated animals. Negative fold change indicates decreased gene induction in RNAi treated animals. Significance was determined with a threshold of p = 0.05.
(DOCX)

Table S6 Compilation of cytoprotective gene expression, lifespan and stress tolerance phenotypes. Twenty-nine genes required for cytoprotective gene induction were characterized for contributions to lifespan extension and stress tolerance. Those that disrupt cytoprotective gene induction, specifically reduce the lifespan of one or more long-lived mutants and are required for xenobiotic stress tolerance are of greatest interest. The most compelling intersections of these phenotypes are observed upon inactivation of *phi-50*, *ima-3*, *elt-2*, *nekl-2*, *wnk-1*, *let-92*, *mdt-26*, and *let-70*. In order to provide an overview of these data, we have compiled all results described throughout the body of this manuscript in a single table.
(XLSX)
