# Supplementary material for: Induction of Cytoprotective Pathways Is Central to the Extension of Lifespan Conferred by Multiple Longevity Pathways
Source: PLoS Genet. 2012 Jul 19;8(7):e1002792. doi: 10.1371/journal.pgen.1002792 (PMC3400582; doi:10.1371/journal.pgen.1002792)
Supplement: Table S1 — Thirteen stress-responsive GFP fusion genes. We analyzed the induction of thirteen stress-responsive GFP fusion genes by gene inactivations that extend longevity. The list encompasses a variety of diverse cytoprotective functions, including unfolded protein responses, innate immunity, oxidative stress response, insulin/IGF-1 signaling and detoxification. This collection of genes and functions represents a useful but circumscribed survey of cytoprotective pathways. *Lamitina T, Huang CG, Strange K (2006) Genome-wide RNAi screening identifies protein damage as a regulator of osmoprotective gene expression. Proc Natl Acad Sci U S A 103: 12173–12178. (DOCX) [file pgen.1002792.s005.docx]

| **Fusion Gene** | **Stress Response** | **Reference** |
| --- | --- | --- |
| p*hsp-6*::gfp | Mt UPR | [[23](#_ENREF_23)] |
| p*hsp-60*::gfp | Mt UPR | [[23](#_ENREF_23)] |
| p*hsp-4*::gfp | ER UPR | [[20](#_ENREF_20)] |
| p*gst-4*::gfp | Detoxification, ROS | [[52](#_ENREF_52)] |
| p*sod-3*::gfp | ROS response | [[53](#_ENREF_53)] |
| p*F55G11.7*::gfp | Innate immunity | [[54](#_ENREF_54)] |
| p*gpdh-1*::gfp | Osmotic stress | Lamitina et al.* |
| p*lys-1*::gfp | Innate immunity | [[54](#_ENREF_54)] |
| p*lys-7*::gfp | Innate immunity | [[54](#_ENREF_54)] |
| p*nlp-29*::gfp | Innate immunity | [[54](#_ENREF_54)] |
| p*fat-7*::gfp | Insulin/IGF-1 signaling | [[32](#_ENREF_32),[55](#_ENREF_55)] |
| *daf-16*::gfp | Insulin/IGF-1 signaling | [[56](#_ENREF_56)] |
| p*hsp-16.2*::gfp | Heat shock | [[57](#_ENREF_57)] |

**Table S1. Thirteen stress-responsive GFP fusion genes**
