# Supplementary material for: Induction of Cytoprotective Pathways Is Central to the Extension of Lifespan Conferred by Multiple Longevity Pathways
Source: PLoS Genet. 2012 Jul 19;8(7):e1002792. doi: 10.1371/journal.pgen.1002792 (PMC3400582; doi:10.1371/journal.pgen.1002792)
Supplement: Table S2 — 160 gene-inactivations known to extend longevity. We analyzed the induction of cytoprotective responses by 160 gene inactivations previously found to extend longevity in high-throughput RNAi screens (Hamilton et al. 2005, Hansen et al. 2005 and Curran and Ruvkun 2007). While not comprehensive, these high-throughput screens are the most comprehensive efforts to globally identify such genes. This circumscribed collection is appropriate to our preliminary experiments because it provides a broad canvas of many gene functions that influence longevity, including insulin/IGF-1 signaling, metabolism and translation. Lifespan extension percentages are compiled from the annotated publications, with the larger percentage lifespan extension shown if the gene is found in more than one publication. 1, Curran and Ruvkun 2007; 2, Hansen et al. 2005; 3, Hamilton et al. 2005. (DOCX) [file pgen.1002792.s006.docx]

| **Gene** | **% Ext.** | **Gene** | **% Ext.** | **Gene** | **% Ext.** | **Gene** | **% Ext.** |
| --- | --- | --- | --- | --- | --- | --- | --- |
| *aco-2*^3^ | 19 | *egl-45*^1^ | 52 | *npa-1*^1^ | 32 | T07A9.8^1,3^ | 24 |
| *age-1*^3^ | 31 | *eif-3.B*^1^ | 51 | *nuo-1*^1^ | 23 | T09A5.8^3^ | 3 |
| *asb-2*^2^ | 46 | *eif-3.F*^1^ | 32 | *nuo-2*^2^ | 42 | T20H4.5^3^ | 23 |
| *asg-2*^3^ | 6 | *erm-1*^1^ | 15 | *nuo-3*^2^ | 32 | T21B10.1^1^ | 17 |
| *atp-2*^1^ | 43 | *ero-1*^1^ | 32 | *nuo-4*^2,3^ | 34 | T27F7.3^1^ | 25 |
| *atp-3*^1,2^ | 45 | F09F7.5^3^ | 4 | *nuo-5*^2^ | 31 | *tag-181*^1^ | 21 |
| *atp-4*^2^ | 33 | F19B6.1^1^ | 35 | *pat-4*^1,2^ | 13 | *tag-300*^1^ | 23 |
| *atp-5*^2^ | 40 | F21H12.1^3^ | 9 | *pat-6*^2^ | 21 | *tag-60*^3^ | 11 |
| B0261.4^3^ | 11 | F25G6.2^1^ | 24 | *pfn-2*^3^ | 10 | *tpa-1*^1^ | 28 |
| B0511.6^1^ | 50 | F26A3.4^1^ | 26 | *phi-37*^1^ | 27 | *ttr-1*^2^ | 14 |
| C01F6.1^3^ | 5 | F26E4.6^1,3^ | 31 | *pos-1*^1^ | 20 | *ubh-4*^3^ | 3 |
| C09B7.2^3^ | 10 | F35D2.3^3^ | 3 | *prx-5*^1^ | 38 | *unc-52*^1^ | 11 |
| C18E9.4^3^ | 4 | F40F8.5^3^ | 3 | R08E3.3^3^ | 11 | *unc-62*^1^ | 38 |
| C26B2.2^3^ | 3 | F43E2.7^1^ | 21 | *rab-10*^2^ | 16 | *unc-83*^3^ | 5 |
| C32H11.1^3^ | 8 | F43G9.1^3^ | 23 | *rha-2*^2^ | 32 | *vha-6*^1^ | 24 |
| C33F10.12^3^ | 13 | F49F1.12^3^ | 5 | *ril-1*^2^ | 32 | W07E6.1^1^ | 31 |
| C36H8.1^3^ | 8 | F53F4.11^1^ | 26 | *ril-2*^2^ | 15 | W09C5.8^3^ | 27 |
| C39F7.2^3^ | 9 | F57B10.3^3^ | 9 | *rnf-5*^3^ | 3 | W09H1.5^3^ | 6 |
| C56G2.1^1^ | 20 | F59B8.2^3^ | 6 | *rps-11*^1^ | 28 | Y105C5B.12^1^ | 27 |
| *cchl-1*^2^ | 36 | F59C6.5^1^ | 19 | *rps-3*^1^ | 32 | Y37D8A.12^3^ | 8 |
| *cco-1*^2,3^ | 61 | *gcy-29*^3^ | 6 | *rps-8*^1^ | 18 | Y39F10C.1^3^ | 11 |
| *cco-2*^2^ | 57 | *gei-15*^1^ | 13 | *sams-1*^2^ | 15 | Y39H10A.6^3^ | 12 |
| *cct-4*^1^ | 28 | *glp-1*^1^ | 33 | *sas-5*^1^ | 30 | Y43F8B.12^3^ | 9 |
| *cct-6*^1^ | 20 | *gpi-1*^2^ | 34 | *scrm-1*^3^ | 8 | Y43h11AL.2^3^ | 5 |
| *ced-3*^1^ | 19 | H06H21.8^3^ | 4 | *sel-5*^1^ | 29 | Y46H3C.6^3^ | 5 |
| *ceh-18*^3^ | 20 | *hrp-1*^1^ | 49 | *sem-5*^1^ | 24 | Y53F4B.23^3^ | 3 |
| *col-93*^3^ | 3 | *htp-3*^1^ | 19 | *set-15*^3^ | 5 | Y54E10BR.4^1^ | 15 |
| *crn-5*^1^ | 41 | *iff-1*^3^ | 10 | *set-9*^3^ | 10 | Y54E5A.7^3^ | 5 |
| *cyc-1*^2^ | 87 | *ifg-1*^1^ | 55 | *sid-2*^3^ | 9 | Y56A3A.19^1^ | 21 |
| D1054.14^1^ | 36 | *inf-1*^1^ | 46 | *sinh-1*^2^ | 21 | Y56A3A.9^3^ | 7 |
| D2030.4^3^ | 9 | *inx-14*^3^ | 6 | *spg-7*^1^ | 22 | Y65B4BR.5^1^ | 17 |
| D2030.9^1^ | 24 | *inx-9*^1^ | 27 | *spt-4*^3^ | 4 | Y71G12B.4^3^ | 5 |
| *daf-2*^1,2^ | 79 | K07H8.1^3^ | 9 | *sre-25*^1^ | 23 | Y71H2AR.2^3^ | 21 |
| *ddl-1*^2^ | 26 | K08E3.5^3^ | 20 | *srh-254*^3^ | 7 | Y75B8A.13^3^ | 10 |
| *ddl-2*^2^ | 11 | K10B4.3^3^ | 9 | *sru-17*^3^ | 14 | Y75B8A.33^3^ | 22 |
| *ddl-3*^2^ | 23 | K10D2.2^3^ | 3 | *srw-99,100*^3^ | 11 | Y92C3A.1^3^ | 8 |
| *dic-1*^1^ | 22 | *maoc-1*^2^ | 30 | *srw-20*^3^ | 7 | ZC132.3^1^ | 26 |
| *drr-1*^2,3^ | 37 | *mcm-2*^1^ | 15 | *str-49*^1^ | 15 | ZK1127.5^1^ | 24 |
| *drr-2*^2^ | 10 | *nas-38*^3^ | 8 | T05A1.4^3^ | 8 | ZK686.2^1^ | 24 |
| E03H12.5^3^ | 8 | *nhr-14*^3^ | 6 | T06G6.4^3^ | 11 | ZK896.7^3^ | 5 |

**Table S2. 160 gene-inactivations known to extend longevity**
