# Supplementary material for: Induction of Cytoprotective Pathways Is Central to the Extension of Lifespan Conferred by Multiple Longevity Pathways
Source: PLoS Genet. 2012 Jul 19;8(7):e1002792. doi: 10.1371/journal.pgen.1002792 (PMC3400582; doi:10.1371/journal.pgen.1002792)
Supplement: Table S3 — Cytoprotective response regulatory genes not discussed within the manuscript. Our primary screen identified 73 candidate stress response suppressors, of which 32 were subsequently quantified and analyzed for secondary phenotypes. Forty-one gene inactivations, however, were not subjected to secondary analysis based upon phenotypic strength or functional annotations suggesting that the gene inactivation resulted in a general disruption of transcription or translation. Data reflects the results of our primary stress response suppression screen. Animals carrying the stress-responsive gene fusions phsp-4::gfp, phsp-6::gfp, psod-3::gfp or pgst-4::gfp were raised to young adulthood and treated with tunicamycin, antimycin, a temperature shift in a daf-2ts mutant background or sodium azide, respectively, as described in methods. Results were scored visually and the fluorescence of each condition was graded on a scale of 0, meaning no decrease in fluorescence, to −4, total loss of fluorescence. Data represents the average of five or more replicates of fifty animals. (DOCX) [file pgen.1002792.s007.docx]

|  | **p*hsp-4::gfp*** | **p*hsp-6::gfp*** | **p*sod-3::gfp*** | **p*gst-4::gfp*** |
| --- | --- | --- | --- | --- |
| *eif-3.F* | -4.0 | ND | 0 | ND |
| *ifg-1/eif-4g* | -4.0 | ND | 0 | ND |
| F11A3.2 | -4.0 | -3.0 | 0 | 0 |
| F54D5.11 | -3.9 | -2.2 | 0 | -3.4 |
| *lrs-1* | -3.3 | 0 | 0 | -2.5 |
| TFIIIB | -3.3 | -1.9 | 0 | 0 |
| *cdk-7* | -3.2 | 0 | 0 | 0 |
| *tba-1,2* | -3.1 | 0 | 0 | 0 |
| *ppp-1* | -3.0 | -1.9 | 0 | 0 |
| T10F2.5 | -3.0 | 0 | 0 | 0 |
| Y19D2B.2 | -2.9 | -2.3 | 0 | -1.2 |
| C01F1.2 | -2.8 | -1.2 | 0 | 0 |
| H27M09.2 | -2.8 | -1.8 | 0 | -2.0 |
| *eif-3.D* | 0 | -3.8 | 0 | -3.9 |
| *eif-3.E* | -1.8 | -3.7 | 0 | -2.7 |
| *dyci-1* | 0 | -3.4 | ND | -1.5 |
| *pab-1* | 0 | -3.3 | 0 | 0 |
| K12D12.4 | -2.2 | -3.1 | -1.3 | 0 |
| *unc-32* | 0 | -3.0 | 0 | 0 |
| D1054.15 | 0 | -2.9 | 0 | 0 |
| *dve-1* | 0 | -2.9 | 0 | 0 |
| T23D8.4 | 0 | -2.8 | 0 | 0 |
| *cel-1* | -2.4 | -2.7 | -1.0 | -1.0 |
| *phi-32* | 0 | -2.7 | -2.0 | 0 |
| W04A4.6 | 0 | -2.7 | -1.8 | 0 |
| W06E11.2 | 0 | -2.6 | 0 | 0 |
| *ddx-23* | 0 | -2.5 | 0 | 0 |
| *dic-1* | 0 | -2.5 | 0 | 0 |
| *snr-7* | 0 | -2.5 | 0 | 0 |
| *xpo-2/imb-5* | 0 | -2.5 | -1.5 | 0 |
| *VPS-32.1,2* | 0 | 0 | -3.5 | 0 |
| Y39C12A.2 | 0 | 0 | -3.3 | -1.1 |
| W04G3.3 | 0 | 0 | -3.0 | 0 |
| H34I24.2 | 0 | -1.2 | -2.8 | -2.4 |
| *dnc-1* | 0 | -1.7 | -2.5 | -2.3 |
| *cdk-9* | 0 | 0 | -2.5 | 0 |
| *set-16* | 0 | 0 | 0 | -3.3 |
| *rsp-3* | 0 | -1.2 | 0 | -2.5 |
| *tba-2,5* | 0 | -1.0 | 0 | -2.5 |

**Table S3. Cytoprotective response regulatory genes not discussed within the manuscript**
