# Supplementary material for: Induction of Cytoprotective Pathways Is Central to the Extension of Lifespan Conferred by Multiple Longevity Pathways
Source: PLoS Genet. 2012 Jul 19;8(7):e1002792. doi: 10.1371/journal.pgen.1002792 (PMC3400582; doi:10.1371/journal.pgen.1002792)
Supplement: Table S4 — Quantification of phsp-16.2::gfp and psur-5::gfp fluorescence. Expression of phsp-16.2::gfp and psur-5::gfp was quantified following gene inactivations that disrupt stress-responsive gene induction. Data control for the possibility that these gene inactivations are not specific to cytoprotective functions and instead function generally in gene expression or transgene silencing. Expression of phsp-16.2::gfp was induced by treatment at 37°C for 1 hour. Inactivation of hsf-1, the known master regulator of heat shock response genes, including hsp-16.2, reduces expression of phsp-16.2::gfp by 10.8 fold (data not shown). Of 29 gene inactivations found to disrupt the induction of cytoprotective genes, 27 did not significantly effect the expression of phsp-16.2::gfp and two, elt-2 and sptl-1, were found to contribute partially to this response. We also measured the expression of a non-stress-induced, constitutively expressed gene fusion, psur-5::gfp, and found that that none of the tested gene inactivations significantly regulate this control. As a result, we conclude that none of the gene inactivations tested contribute to transgene silencing or abrogate gene induction. Significance was determined with a threshold of p = 0.05. (DOCX) [file pgen.1002792.s008.docx]

|  | p*hsp-16.2*::gfp | p*sur-5*::gfp |
| --- | --- | --- |
|  | Fold ∆ | Fold ∆ |
| *dcp-66* | 1.2 | 0.9 |
| *sptl-1* | 3.0* | 1.4 |
| *gob-1* | 1.0 | 0.9 |
| *ufd-1* | 1.0 | 0.8 |
| *mdt-26* | 1.5 | 1.2 |
| *sdc-2* | 1.1 | 0.9 |
| F53F4.11 | 1.1 | 0.9 |
| *pas-3* | 1.0 | 1.1 |
| *arf-3* | 1.0 | 1.1 |
| *let-70* | 1.4 | 1.3 |
| *phi-50* | 1.6 | 1.2 |
| *kin-1* | 1.0 | 1.3 |
| Y50D7A.11 | 1.0 | 1.2 |
| *skr-1* | 1.0 | 1.0 |
| *cpf-2* | 1.0 | 1.2 |
| *ima-3* | 1.0 | 1.1 |
| *cpsf-4* | 1.0 | 1.2 |
| *cpsf-2* | 1.5 | 1.3 |
| *daf-16* | 1.0 | 0.9 |
| *ire-1* | 1.0 | 0.9 |
| F18F11.5 | 1.3 | 1.3 |
| *wnk-1* | 1.0 | 1.0 |
| *skn-1* | 1.0 | 1.0 |
| *lin-40/egr-1* | 1.3 | 1.0 |
| *rab-10* | 1.0 | 1.2 |
| C06A8.2 | 1.0 | 1.0 |
| *cul-1* | 1.1 | 1.1 |
| *elt-2* | 2.1* | 1.4 |
| *hda-1* | 1.2 | 1.2 |
| *dpy-22* | 1.0 | 1.1 |
| *let-92* | 1.2 | 1.4 |
| *nekl-2* | 1.3 | 1.1 |

**Table S4. Quantification of p*hsp-16.2*::gfp and p*sur-5*::gfp fluorescence**
