# Supplementary material for: Induction of Cytoprotective Pathways Is Central to the Extension of Lifespan Conferred by Multiple Longevity Pathways
Source: PLoS Genet. 2012 Jul 19;8(7):e1002792. doi: 10.1371/journal.pgen.1002792 (PMC3400582; doi:10.1371/journal.pgen.1002792)
Supplement: Table S5 — qPCR results. Expression of endogenous stress-responsive loci was measured by qPCR to verify phenotypes observed using fluorescent fusion genes. Our primary screen identified gene inactivations required for the induction of phsp-4::gfp, phsp-6::gfp, psod-3::gfp and pgst-4::gfp upon treatment with inducing stimuli. Wild-type (N2) animals with no transgenes were raised and treated under the conditions of our primary screen (see methods) and animals were harvested from each condition with 4 replicates of approximately 4,000 worms per replicate. Expression of cytoprotective genes was quantified for animals fed an empty-vector (L4440) RNAi control or gene inactivations identified in the stress response suppression screen. Inducing conditions were applied to both vector-fed and RNAi-fed animals. GFP data represents the fold decrease fluorescence as compared to the empty-vector control and is identical to data from Table 1. qPCR data are presented as the difference in fold induction between vector and RNAi treated animals. Negative fold change indicates decreased gene induction in RNAi treated animals. Significance was determined with a threshold of p = 0.05. (DOCX) [file pgen.1002792.s009.docx]

| Gene | (RNAi) | GFP | qPCR | qPCR StDev |
| --- | --- | --- | --- | --- |
| *sod-3* | *daf-16* | 96.3 | -9.5 | 1.2 |
|  | *elt-2* | 3.2 | -8.3 | 0.3 |
|  | *let-70* | 4.7 | -5.8 | 2.5 |
|  | *gob-1* | 3.2 | -5.2 | 2.8 |
|  | *phi-50* | 5.8 | -5.2 | 2.5 |
|  | *arf-3* | 1.5 | -4.3 | 2.7 |
|  | *dcp-66* | 13.9 | -4.3 | 3.0 |
|  | *let-92* | 3.2 | -4.1 | 3.4 |
|  | *lin-40/egr-1* | 17.2 | -3.2 | 1.4 |
|  | *hda-1* | 5.9 | -2.3 | 0.3 |
|  | *dpy-22* | 1.6 | -2.2 | 0.5 |
|  | *sdc-2* | 2.5 | -1.0 | 4.9 |
|  | *kin-1* | 2.0 | 2.6 | 0.3 |
| *gst-4* | *skn-1* | 2.2 | -13.9 | 3.4 |
|  | *skr-1* | 1.5 | -3.7 | 3.6 |
|  | *rab-10* | 1.8 | -3.0 | 0.6 |
| *hsp-6* | *let-70* | 31.6 | -3.8 | 7.4 |
|  | *cpsf-4* | 3.9 | -3.0 | 10.6 |
|  | *sptl-1* | 3.9 | -2.9 | 0.6 |
|  | *elt-2* | -21.7 | -2.2 | 1.8 |
|  | *let-92* | 3.9 | -1.7 | 1.8 |
|  | Y50D7A.11 | 1.5 | -0.1 | 4.4 |
|  | *pas-3* | 2.7 | -0.1 | 1.8 |
|  | *ufd-1* | 1.7 | 0.0 | 0.4 |
|  | *cpf-2* | 2.7 | 0.1 | 4.2 |
|  | *rab-10* | 2.6 | 0.3 | 0.1 |
| *hsp-4* | *ire-1* | 13.0 | -5.8 | 6.9 |
|  | Y50D7A.11 | 2.5 | -5.6 | 5.3 |
|  | *ima-3* | 3.8 | -4.1 | 6.6 |
|  | *mdt-26* | 3.2 | 0.4 | 1.3 |
|  | *let-70* | 9.7 | 0.9 | 11.0 |

**Table S5. qPCR results**
